# Age-Related Cognitive Bias in the Assessment of Lateral Pinch Modulation by Occupational Therapists

**DOI:** 10.3390/s23187747

**Published:** 2023-09-08

**Authors:** Naama Karniel, Eden Shimon, Noam Gemer, Rotem Zivion, Sigal Portnoy

**Affiliations:** 1 Hadassah Medical Center, Department of Physical Therapy, Jerusalem 9765418, Israel; dkar435@hadassah.org.il; 2 Department of Occupational Therapy, Faculty of Medicine, Tel Aviv 6997801, Israel; eden951s@gmail.com (E.S.); noammmm78@gmail.com (N.G.); rotemzivion9304@gmail.com (R.Z.)

**Keywords:** occupational therapy, Rational-Experiential Inventory-40, cognitive reflection test

## Abstract

Background: Cognitive bias may appear in occupational therapists’ interpretation of physical examinations. Since different strategies for decision making have been shown to reduce bias, its quantification is an essential first step towards awareness and bias reduction. Our aims: (1) quantify cognitive bias by testing the differences in occupational therapists’ assessment of lateral pinch force modulation between young and older adults, and between women and men; and (2) to test for a correlation between the tendency to bypass an intuitive response and the degree of cognitive bias. Methods: Occupational therapists (n = 37; age 40.3 ± 11.4 years) used a visual analogue scale to rate pre-recorded simulations of the digital output of lateral pinch modulation videos of different levels of abilities coupled with videos of young/old men/woman pressing the force sensor. They filled out the Cognitive Reflection Test and the Rational-Experiential Inventory-40. Results: Subjects showed higher bias towards old individuals compared to young ones (*p* < 0.001), but with no sex bias (*p* = 0.119). Rational ability correlated with cognitive bias of assessment of lateral pinch modulation in old individuals (r = 0.537, *p* < 0.001). Discussion: Occupational therapists might underestimate the physical abilities of older adults. Biased evaluation might cause assignment of redundant exercises and therefore loss of time, effort, and resources.

## 1. Introduction

Cognitive bias, characterized by systematic thought patterns that can introduce errors in memory, perception, or judgment [1], has accumulated extensive attention within decision-making contexts. The process of decision making is governed by a dual reasoning mechanism, encompassing intuitive rapid processing and a more deliberate, complex cognitive process [2,3]. Although these mechanisms function concurrently, one may exert dominance and contribute to cognitive bias. Notably, research has indicated that the ability to counteract intuitive responses, an attribute that can be measured by tools like The Cognitive Reflection Test, can mitigate cognitive biases [2]. This suggests that awareness of these underlying factors holds potential for enhancing decision quality.

Cognitive bias has been well-documented across various medical domains and is implicated at all stages of clinical diagnosis, including anamnesis and prognosis [4]. Age and sex biases among rehabilitation clinicians have been demonstrated through case descriptions and attitudinal assessments [5]. Despite this extensive documentation in medicine, a scarcity of literature addresses bias in physical assessments. In fact, a recent review highlighting cognitive biases across diverse fields revealed a scarcity in research within health professions [6]. Among 149 articles related to cognitive bias in health professions, the majority featured psychologists, with only a single study involving occupational therapists. Many of the existing biases documented in the medical field are derived from case studies in which clinicians make decisions based on patient evaluation files. However, a crucial consideration arises: what if biases are already present within these evaluations due to the potential bias of the occupational therapist conducting the physical assessment? This area remains unexplored.

To the best of our knowledge, there is only one research that tested for bias during the process of actually evaluating physical properties. The study involves physiotherapists measuring wrist range of motion [7]. A group with no prior patient information measured an angle of 80.2 ± 1.8°, while a group influenced by biased patient history data recorded a significantly reduced angle of 72.4 ± 1.8°. This study showcases the impact of bias on physical assessment that may result in underestimation of the abilities of the patient and compromise the patient’s wellbeing. Our study aims to quantitatively assess cognitive bias in evaluating lateral pinch force modulation through observational rating. Specifically, our objectives include (1) discerning differences in occupational therapists’ assessments of lateral pinch force modulation between young and older adults; (2) investigating disparities in occupational therapists’ assessments based on gender; and (3) exploring correlations between the inclination to counteract intuitive responses and the extent of cognitive bias.

## 2. Materials and Methods

Population: We recruited 37 occupational therapists (36 females, mean and standard deviation of age 40.3 ± 11.4 years) using a convenient and snowball sampling method by referring to clinics across Israel, employing manual occupational therapists in the physical or geriatric field. The inclusion criteria were at least one year of experience in the physical or geriatric field, with normal or corrected eyesight and hearing. The participants had 13.4 ± 12.2 years of clinical experience in the physical field. Thirty-four (91.9%) participants had experience working with the geriatric population. The University institutional review board granted ethical approval (#0002681-1). All of the participants read and signed an informed consent form pretrial.

Tools: In this study, we collected data regarding the tendency of the participants to bypass an intuitive response, as well as their rational information processing and experiential information processing. In order to test the bias of the participants in evaluating lateral pinch modulation of individuals of different sexes and ages, we coupled pre-recorded simulation videos of the digital output of pinch examinations of different levels of abilities, with videos of male and female, old (above 65 years old) and young (in their 20s) individuals pressing the force sensor. These tools are detailed below.

The Cognitive Reflection Test (CRT) [8] assesses the inclination to avoid a fast, incorrect response. The test consists of three questions of different difficulty levels. The scores range from ‘0’ (low ability to bypass an intuitive response) to ‘3’ (high ability to bypass an intuitive response). The CRT has substantial correlations with common biases in judgments and decisions [9]. Therefore, it can shed light on whether therapists’ cognitive biases arise from more analytical, conscious thought processes (rational processing) or from intuitive, pre-conscious responses (experiential processing). The tool has moderate validity [10] and moderate internal reliability [3].

The Rational-Experiential Inventory-40 (REI-40) [11] is a subjective 40-item questionnaire that rates responses on a scale from ‘1’ (definitely not true) to ‘5’ (definitely true). This tool measures two modes of information processing: rational and experiential [11]. Rational processing emphasizes analytical, conscious thinking, as seen in items like “I usually have clear, explainable reasons for my decisions”. Experiential processing is more intuitive and affective, as in “I believe in trusting my hunches”. An average of 10 items generates subscale scores for the rational ability, rational engagement, experiential ability, and experiential engagement of each participant. Thus, each respondent receives scores for rational ability, rational engagement, experiential ability, and experiential engagement [11]. This inventory enables the examination of the therapists’ cognitive tendencies and approaches when forming clinical judgments. The REI-40 has high internal reliability [12].

**Figure 1:**
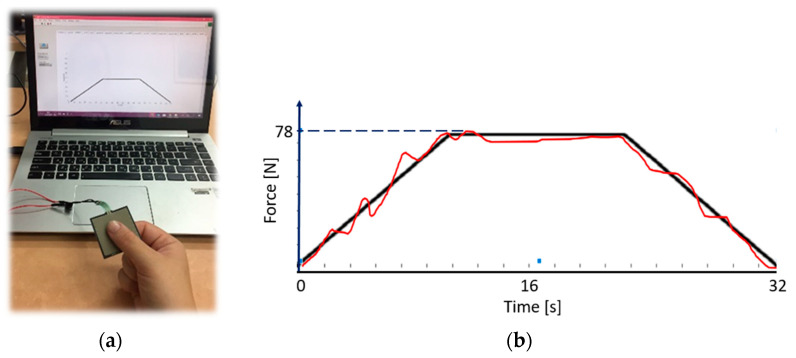
(**a**) A square force sensor that controls (**b**) a virtual moving red force line, the height of which is determined by the extent of the lateral pinch force. A black constant ramp line serves as the pattern for the red line tracing. The red line is moving from left to right so that it reaches the end of the ramp in 32 s.

We used a system for evaluating the modulation of the lateral pinch force. We chose the physical measure of force modulation, since conventional evaluation, e.g., grip force, provides a singular measurement that can be accurately evaluated, as it produces a final value of measurement. The system (Figure 1a) consists of a 3.8 × 3.8 cm square sensor (Interlink electronics, Irvine, CA, USA), which measures force between 0.981 N and 98.1 N. Virtual feedback is provided on screen on which a red graph is displayed, the height of which is controlled by the amount of force applied by the participant. The participant is asked to follow the fixed trajectory of a ramp while the red force line is moving from left to right, so that it reaches the end of the ramp in 32 s (Figure 1b). 

The raw score was calculated as the root mean square error (RMSE) between the fixed ramp points, *y_i_*, and the produced trajectory points, *y_j_*, for a number of *n* points, according to the following formula:

(1) $$RMSE=\sqrt{\frac{\sum {\left(y_{i}-y_{j}\right)}^{2}}{n}}$$

**Figure 2:**
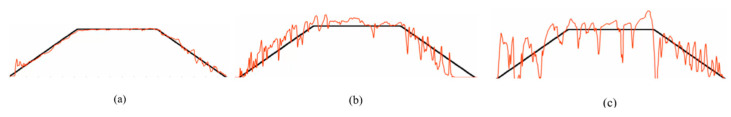
Digital recording of the lateral pinch modulation tests: (**a**) good force modulation (normalized score = 1.6), (**b**) moderate force modulation (normalized score = 4.6), (**c**) bad force modulation (normalized score = 9.0). A black constant ramp line serves as the pattern for the red line tracing. The red line is moving from left to right so that it reaches the end of the ramp in 32 s. The amount of actual force applied is not shown during assessment, to prevent further bias by the assessors.

In order to make the scoring process more intuitive, the raw RMSE score was normalized using an RMSE value produced when the sensor is not pressed, i.e., an extremely high error, marked as max RMSE, according to the following formula:

(2) $$Normalized\;score=\frac{normalized\;score\;range}{\max RMSE}\cdot raw\;score+1$$

where the normalized score range is 9 (from ‘1’ to ‘10’). A perfect alignment produces an error score of ‘1’. The highest error produces an error score of ‘10’. We performed pre-recordings of various performances and divided them into good (scores 1–3), moderate (scores 4–7), and bad (scores 8–10) performances (examples in Figure 2). Since we wanted to make sure that for each patient category, e.g., an elderly woman, the subjects viewed a range of performances (from bad to good), and that this performance range was also similar to that shown for the other three patient categories, i.e., an elderly man and a young man and woman. Given that each category had three patients, we omitted intermediate scores that fell between the “good” and “moderate” or “moderate” and “bad” classifications. This approach maintained comparability between categories, while focusing on distinct performance levels.

The Visual Analogue Scale (VAS) [13] is represented by a 10 cm horizontal line. The participants are asked to mark their subjective level regarding a question. The VAS is used for a variety of purposes, including subjective assessment of performance by observation [14]. In the field of physical training, VAS was used by participants, who are coaches, to evaluate performance of their trainees in basketball [14] or rugby [15], in which videos of the performance were used, as in our study. In the present study, the participants were asked to rate their assessment of patients’ lateral pinch modulation. The participants also rated their level of confidence after each assessment. The VAS has medium-high reliability [16] and structure validity [17].

**Figure 3:**
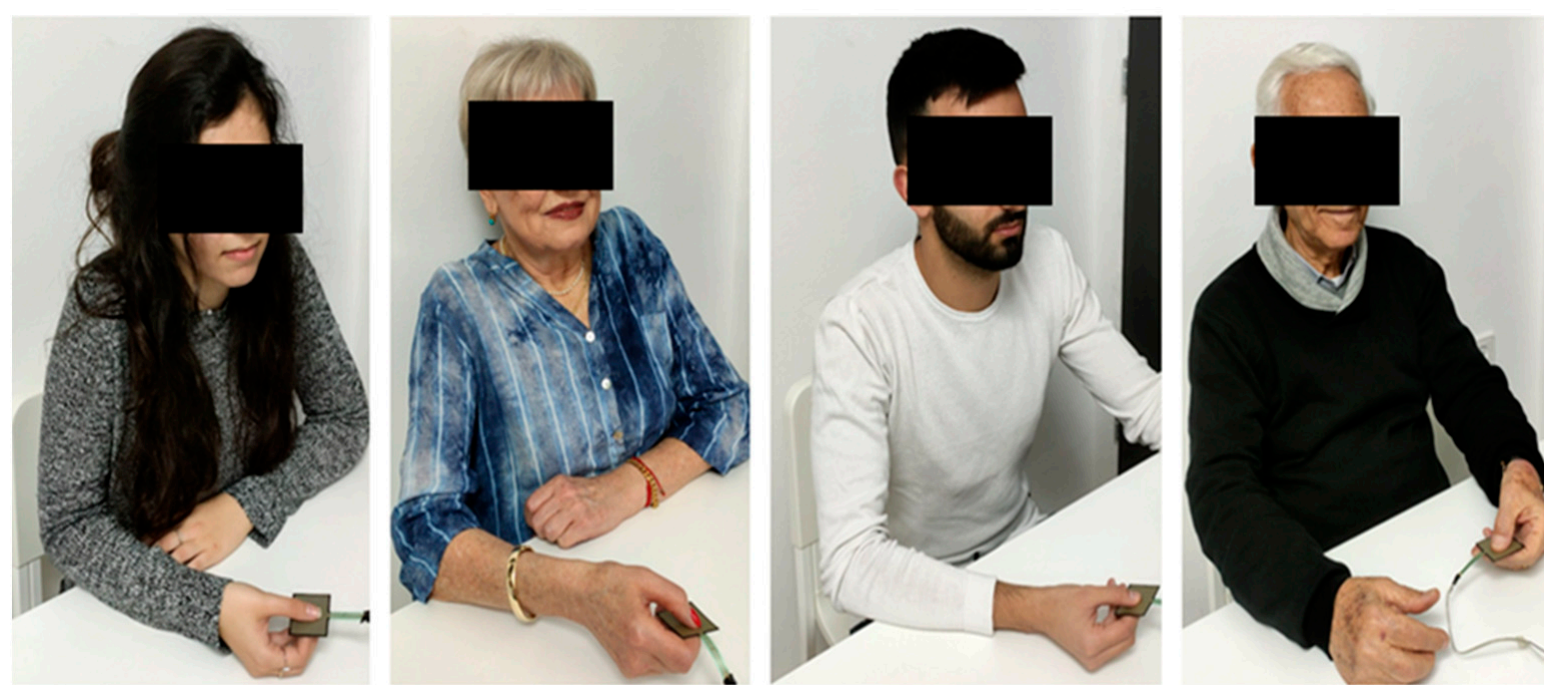
Examples of videos showing patients holding the force sensors. From left to right: young female, old female, young male, old male. The subjects were shown these videos without the eye masking.

Protocol: The participant received an explanation regarding the force modulation evaluation process. The participant viewed three video recordings of the digital output of good, moderate, and bad performances (no patients were seen in these videos). The score was provided and explained. Then, for the learning process, the participants were presented with ten videos of the digital output of good, moderate, and bad performances (no patients were seen in these videos). The participants were ask to rate the performance and then the score was presented to them. The mean and standard deviation error for the learning evaluations was −0.2 ± 0.4. Then, the participants were asked to rate 16 video recordings of the digital output, where a patient holding the sensor was seen, adjacent to the graphic output. We artificially coupled the videos of three old males with videos of good, moderate, and bad performances. Similarly, we coupled videos of various performances with videos of three old females, three young males, and three young females (Figure 3). Videos were presented randomly. Finally, the participants filled out the REI-40 and CRT questionnaires.

Statistical analysis: Statistical analyses were performed using SPSS 27.0 (SPSS Chicago, IL, USA). Statistical significance was considered at *p* < 0.05. The bias for young, old, male, or female was calculated as the difference between the actual score and the score given by the participant, averaged for the six videos, as follows:

(3) $$Bias=\frac{\sum_{6}\left(assessment\;score-normalized\;score\right)}{6}$$

Score of ‘0’ means no bias. A positive score means that the performance was estimated to be worse than it actually was (the subject gave a higher normalized score than the actual calculated score), while a negative score means that the performance was estimated to be better than the actual performance.

We employed the Shapiro–Wilk test to assess normal distribution. Due to the non-normal distribution of certain outcome measures, non-parametric tests were subsequently utilized. The Wilcoxon test was used due to the small sizes of the groups. The effect size, *r*, was calculated using the following equation [18]:

(4) $$r=\frac{Z}{\sqrt{N}}$$

Correlations were performed using the Spearman correlation test. 

## 3. Results

**Figure 4:**
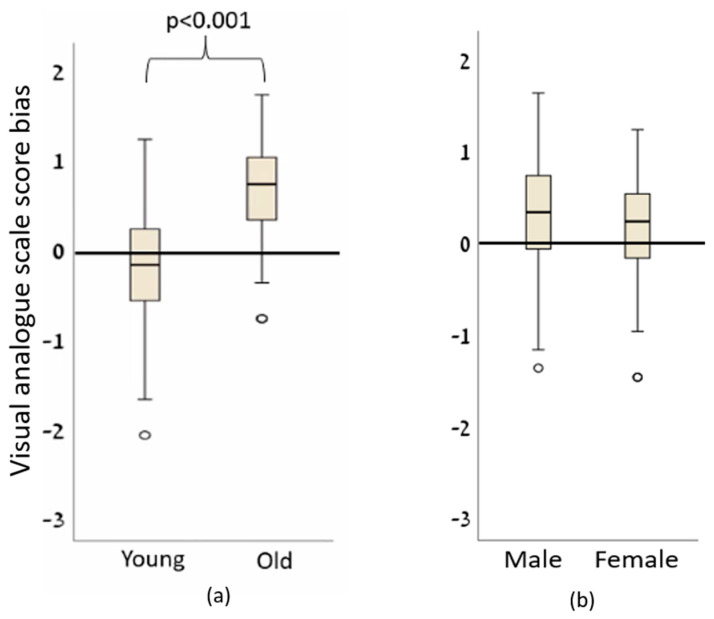
The bias of the subjects’ assessment of the performance of (**a**) old and young people and (**b**) female and male. A score of ‘0’ means no bias. A positive score means that the performance was estimated to be worse than it actually was (the subject gave a higher normalized score than the actual calculated score), while a negative score means that the performance was estimated to be better than the actual performance.

We found statistically significant higher bias towards old individuals compared to young ones (*p* < 0.001; Figure 4a; Table 1). There were no differences in the bias between male and female individuals (*p* = 0.119; Figure 4b; Table 1). We used G*Power (version 3.1.9.6, Kiel University, Düsseldorf, Germany) to calculate the power of our findings. For our sample size of 37, with an alpha of 0.05, the power calculated for the age bias is 100%, and for the sex bias 21.5%. This indicates that our research design provides a very strong likelihood of identifying significant age-related effects that might be present in our data. However, the low power for detecting sex bias suggests that with our sample size and study design, reliable detection of sex-related effects might be insufficient, potentially leading to the omission of true effects.

Although there were statistically significant differences in the confidence levels for evaluating males versus females and old versus young individuals (Table 1), the differences between the medians were very small (0.1).

**Figure 5:**
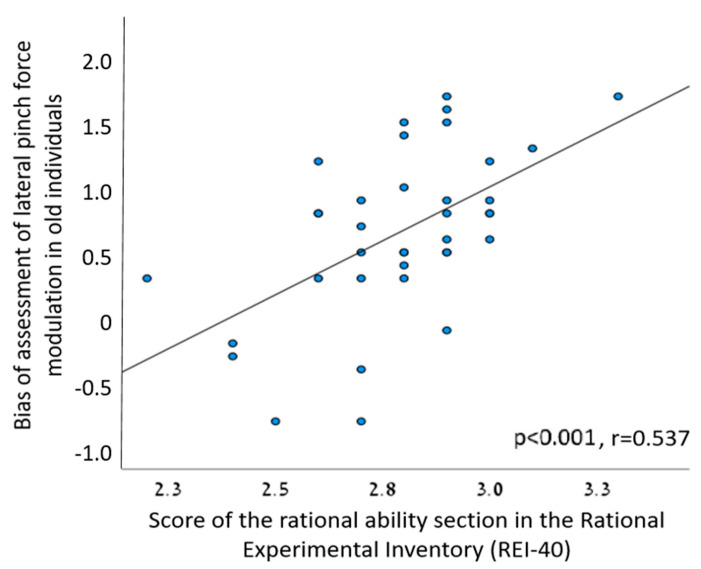
A significant positive correlation between the score of the rational ability section in the REI-40 and the cognitive bias of assessment of lateral pinch force modulation in old individuals.

We found a statistically significant correlation between the score of the rational ability section in the REI-40 and the cognitive bias of assessment of lateral pinch force modulation in old individuals (Figure 5, Table 2). There was no statistically significant correlation between the CRT scores and age-related cognitive bias (Table 2).

## 4. Discussion

We found age-related, but not sex-related, cognitive bias, to be evident when occupational therapists assessed lateral pinch modulation. Additionally, higher bias for older individuals correlated with higher scores for rational information processing. No correlation was found between the tendency to bypass an intuitive response and the degree of cognitive bias.

While our study is the first to document bias within a dynamic clinical assessment, our findings align with prior literature that has explored the broader aspects of clinical decision-making. For example, age bias was reported in a study of 974 rehabilitation professionals [5], introduced with identical case studies that differed only in the age/sex of the patients. The participants estimated lower rehabilitation potential for the older patients, without sex bias. Our study diverges from the previously mentioned research by adopting an alternative approach. Instead of presenting participants with a story lacking the visual context of the patient, we introduced a video presentation that integrates the patient’s actions alongside a performance, presented as the patient’s performance. This innovative method may yield distinct outcomes, as we solicit consensual judgments from seeing a face [19], which could potentially influence the magnitude of bias observed. Furthermore, our study contributes a novel perspective by being the first to uncover the existence of bias in the evaluation of a patient’s physical abilities. This unexplored aspect sheds light on the potential influence of cognitive biases in clinical assessments, extending the current understanding of decision-making dynamics within healthcare contexts. Although data regarding age bias in health profession is scarce, the current literature on age bias in medicine is vast. For example, 87% of 204 rheumatologists preferred aggressive drugs for the treatment of a hypothetical patient with rheumatoid arthritis that was presented to them as a 28-year-old patient, but only 71% preferred that drug when the patient was presented as an 82-year-old patient [20]. Our study centers on quantitative physical tests, distinguishing it from the aforementioned studies which focused on patient prognosis evaluations. This novelty is significant as it shifts the focus from subjective prognostic assessments to objective measurements of physical capabilities. By addressing a quantitative aspect of patient evaluation, our study contributes to a more comprehensive understanding of the potential biases that can impact clinical decisions, thereby enhancing the applicability of our findings to real-world healthcare scenarios.

The literature underscores the population variability in measures of gross motor competence across sexes and age groups [21]. Notably, while sex-related athletic performance differences are evident in sports, with males often outperforming females by an average of 10% [22], physiological distinctions are also evident. Males tend to exhibit greater muscle mass and strength, along with superior aerobic capacity, while females display attributes such as reduced muscle fatigability and enhanced recovery during endurance exercise [23]. Given these insights, one might anticipate that women possess superior manual dexterity compared to men. However, the scarcity of research focused on sex-related variations in force modulation complicates such predictions. Furthermore, the existing literature surprisingly indicates no sex-based differences in manual dexterity [24]. Our interpretation of our findings rests on multiple considerations. First, the limited reports examining the force modulation abilities of men compared to women may underlie the absence of cognitive bias among our participants. Alternatively, the nuanced interplay of physiological, cognitive, or task-specific factors might collectively contribute to this outcome. While the prospect of differing results with a larger male participant group is plausible, it is equally pertinent to consider the multifaceted nature of cognitive biases in clinical assessments. Noteworthy is a previous study highlighting the differential assessments made by male and female physicians in similar cases involving patients of both sexes [25]. Our findings intersect with this broader body of literature, underscoring the intricate dynamics of sex-related biases in medical contexts. In conclusion, the absence of sex-related bias in force modulation assessments presents an intriguing avenue for further exploration, offering potential insights for refining clinical decision-making practices.

The observed positive correlation between the scores in the rational ability section of the REI-40 and the cognitive bias associated with the assessment of lateral pinch force modulation among older individuals provides intriguing insights into the interplay of cognitive biases and self-perceived rationality. The finding implies that individuals who perceive themselves as more rational tend to exhibit a stronger bias toward assessing older patients, indicating a potential connection between cognitive biases and subjective assessments of rationality. Notably, the absence of prior research linking bias to self-reported rational ability underscores the novelty of this finding. Our study introduces a unique perspective by shedding light on the potential influence of decision-making strategies driven by rational cognition. We hypothesize that individuals who exhibit a rational decision-making approach might heavily rely on statistical analyses of past experiences when compared to their more intuitive counterparts. In the context of therapeutic decision-making, it is plausible that rational therapists lean more heavily on accumulated knowledge regarding the prevalent physical decline observed in older adults as opposed to younger ones. This could potentially contribute to the emergence of biased judgments. The nuanced interplay between rational thinking, cognitive biases, and their impact on decision-making processes in clinical settings warrants further exploration. This discovery underscores the complexity of decision-making processes within the medical domain and prompts the consideration of a broader range of factors that can influence bias in clinical assessments. Future investigations could delve into the specific cognitive mechanisms through which self-perceived rationality interacts with biases, enriching our understanding of how these factors collectively shape clinical judgments and patient outcomes.

The study of cognitive bias in medicine, and specifically in health professions, is an imperative step towards understanding the effects of these biases on clinical decision making. The main finding of our study, i.e., under-estimation of pinch modulation abilities in older adults, may be interpreted by clinicians as sensory deficits affecting pinch, as seen, for example, during pinch grip in individuals post-stroke [26]. A possible result of the biased evaluation of an older adult, as found in our study, might have been the assignment of redundant exercises for fine motor skills combined with specific intrinsic and extrinsic hand muscle-strengthening exercises. This would have required needless resources of time, effort, and costs from the patient and health provider. For example, resistance, steadiness, and functional training are often prescribed to older adults to improve motor control [27]. In order to minimize this effect, various measures should be considered. Importantly, previous reports have shown the efficacy of interventions in reducing biases by using different strategies for making decisions. For example, we may promote awareness of the existence of biases and exercise critical thinking [28,29] by using simulations to learn about the subject [30]. Awareness of the existence of biases is the first step to overcoming cognitive biases [4]. The principle of intervention and practice program to reduce biases was found to be the most effective means to improve awareness and reduce biases in decision-making among physicians [31]. Therefore, interventions to increase awareness of bias are recommended in order to reduce biases in health professionals. Further evidence has shown a reduction in biases following a program of three sessions in physicians [31], and also in medical students who received a one-time intervention of approximately 20 min [32]. However, such an intervention has never been tested in health profession practitioners. Another possible solution for reducing bias might be usage of objective and precise measurements of physical abilities. As new technologies become widely available [33], occupational therapists should strive to incorporate them into patient evaluation. 

The present study has several limitations. First, the study was conducted among female occupational therapists. Although the inclusion criteria were not limited to females, male occupational therapists are few. Therefore, our findings cannot be generalized to the entire population of therapists of male clinicians (occupational therapists, physiotherapists, etc.). The behaviors, decision-making patterns, and cognitive biases of male therapists might differ from their female counterparts due to a myriad of factors, including socialization, training, and individual experiences. As a result, any conclusions drawn from the study’s findings may not accurately represent the entire spectrum of clinicians. With that said, since the U.S. Census statistics [34] show that the workforce of occupational therapists in 2017 consisted of 87.1% women, our results are relevant to this great majority of clinicians, and best represent occupational therapists demographics. Second, due to the limitations of the COVID-19 pandemic, the study was conducted using “Zoom” software on a computer, as the participants watched the videos. 

## 5. Conclusions

In conclusion, occupational therapists might not be aware of their bias in interpreting physical examinations of older adults. This finding, along with the accumulating evidence of ageism in medicine, supports the WHO’s recent recognition of ageism as a public health issue and as one of the most prevalent forms of stereotyping, prejudice and discrimination [32]. We therefore believe that more research is needed to collect further evidence of biases in health professionals and various physical examinations, e.g., observational gait analysis. It would be interesting to investigate how biases vary across different cultural contexts and years of experience. Additionally, a longitudinal study should investigate how biases in physical assessments impact patient outcomes and treatment efficacy. Regarding bias prevention, the efficacy of intervention programs in teaching therapists to be aware and reduce bias, possibly using simulation training and objective assessment tools, should be studied. Further knowledge of the effect of age bias and means to prevent it could help reduce the unnecessary spending of clinician and patient’s time and funds. 

## Figures and Tables

**Table 1:** The median and interquartile range (IQR) of the mean bias (a positive value means worse modulation ability) and confidence in the evaluation of young and old females and males. The *p* values and effect size, *r*, are presented.

		Median and IQR	*p*	*r*
Mean bias	Old	0.7 (0.3–1.1)	<0.001	−0.857
	Young	−0.20 (−0.75–0.25)		
Confidence (1–10)	Old	8.2 (7.2–9.0)	0.019	−0.386
	Young	8.1 (7.3–8.7)		
Mean bias	Male	0.3 (−015–0.75)	0.119	−0.256
	Female	0.2 (−0.2–0.5)		
Confidence (1–10)	Male	8.2 (7.0–9.0)	0.021	−0.378
	Female	8.1 (7.4–8.9)		

**Table 2:** Engagement between age-related cognitive bias and scores of the Rational-Experiential Inventory-40 and the cognitive reflection test.

		*p*	*r*
Rational-Experiential Inventory-40	Rational ability	0.001	0.537
	Rational favorability	0.568	0.097
	Experiential ability	0.938	−0.013
	Experiential favorability	0.440	0.131
The Cognitive Reflection Test		0.750	−0.054

## Data Availability

Portnoy, Sigal (2023), “Karniel”, Mendeley Data, V2. https://doi.org/10.17632/crgydzc8g8.2. (accessed on 5 September 2023).

## References

[1] Tversky A., Kahneman D. (1974). Judgment under Uncertainty: Heuristics and Biases. Biases in Judgments Reveal Some Heuristics of Thinking under Uncertainty. Science.

[2] Stanovich K.E., West R.F. (2000). Individual Differences in Reasoning: Implications for the Rationality Debate?. Behav. Brain Sci..

[3] Szaszi B., Szollosi A., Palfi B., Aczel B. (2017). The Cognitive Reflection Test Revisited: Exploring the Ways Individuals Solve the Test. Think. Reason..

[4] O’Sullivan E.D., Schofield S.J. (2018). Cognitive Bias in Clinical Medicine. J. R. Coll. Physicians Edinb..

[5] Rybarczyk B., Haut A., Lacey R.F., Fogg L.F., Nicholas J.J. (2001). A Multifactorial Study of Age Bias among Rehabilitation Professionals. Arch. Phys. Med. Rehabil..

[6] Featherston R., Downie L.E., Vogel A.P., Galvin K.L. (2020). Decision Making Biases in the Allied Health Professions: A Systematic Scoping Review. PLoS ONE.

[7] Rotem-Lehrer N., Singer N., Reshit O., Springer S. (2016). Measuring up to Expectation: Cognitive Bias in Wrist Range-of-Motion Measurement. J. Orthop. Sports Phys. Ther..

[8] Frederick S. (2005). Cognitive Reflection and Decision Making. J. Econ. Perspect..

[9] Baron J., Scott S., Fincher K., Emlen Metz S. (2015). Why Does the Cognitive Reflection Test (Sometimes) Predict Utilitarian Moral Judgment (and Other Things)?. J. Appl. Res. Mem. Cogn..

[10] Toplak M.E., West R.F., Stanovich K.E. (2011). The Cognitive Reflection Test as a Predictor of Performance on Heuristics-and-Biases Tasks. Mem. Cogn..

[11] Keaton S.A. (2017). Rational-Experiential Inventory-40 (REI-40). The Sourcebook of Listening Research.

[12] Björklund F., Bäckström M. (2008). Individual Differences in Processing Styles: Validity of the Rational-Experiential Inventory. Scand. J. Psychol..

[13] Gift A.C. (1989). Visual Analogue Scales:Measurement of Subjective Phenomena. Nurs. Res..

[14] Baghurst T., Lackman J., Drewson S., Spittler P., Turcott R., Smith M., Illescas-Marquez G., Boolani A. (2021). A Hot Mess: Basketball Coaches’ Perceptions of Ability versus Actual Performances of Their Athletes. Auc Kinanthropologica.

[15] Campo M., Champely S., Lane A.M., Rosnet E., Ferrand C., Louvet B. (2019). Emotions and Performance in Rugby. J. Sport Health Sci..

[16] Boonstra A.M., Schiphorst Preuper H.R., Reneman M.F., Posthumus J.B., Stewart R.E. (2008). Reliability and Validity of the Visual Analogue Scale for Disability in Patients with Chronic Musculoskeletal Pain. Int. J. Rehabil. Res..

[17] Brokelman R.B.G., Haverkamp D., van Loon C., Hol A., van Kampen A., Veth R. (2012). The Validation of the Visual Analogue Scale for Patient Satisfaction after Total Hip Arthroplasty. Eur. Orthop. Traumatol..

[18] Strack F. (1992). “Order Effects” in Survey Research: Activation and Information Functions of Preceding Questions. Context Effects in Social and Psychological Research.

[19] Zebrowitz L.A., Franklin R.G., Hillman S., Boc H. (2013). Older and Younger Adults’ First Impressions From Faces: Similar in Agreement but Different in Positivity. Psychol. Aging.

[20] Fraenkel L., Rabidou N., Dhar R. (2006). Are Rheumatologists’ Treatment Decisions Influenced by Patients’ Age?. Rheumatology.

[21] Puh U. (2010). Age-Related and Sex-Related Differences in Hand and Pinch Grip Strength in Adults. Int. J. Rehabil. Res..

[22] Barnett L.M., Lai S.K., Veldman S.L.C., Hardy L.L., Cliff D.P., Morgan P.J., Zask A., Lubans D.R., Shultz S.P., Ridgers N.D. (2016). Correlates of Gross Motor Competence in Children and Adolescents: A Systematic Review and Meta-Analysis. Sports Med..

[23] Bassett A.J., Ahlmen A., Rosendorf J.M., Romeo A.A., Erickson B.J., Bishop M.E. (2020). The Biology of Sex and Sport. JBJS Rev..

[24] Haward B.M., Griffin M.J. (2002). Repeatability of Grip Strength and Dexterity Tests and the Effects of Age and Gender. Int. Arch. Occup. Environ. Health.

[25] Hamberg K., Risberg G., Johansson E.E. (2004). Male and Female Physicians Show Different Patterns of Gender Bias: A Paper-Case Study of Management of Irritable Bowel Syndrome. Scand. J. Public Health.

[26] Blennerhassett J.M., Matyas T.A., Carey L.M. (2007). Impaired Discrimination of Surface Friction Contributes to Pinch Grip Deficit after Stroke. Neurorehabil. Neural Repair.

[27] Fiogbé E., Carnavale B.F., de Medeiros Takahashi A.C. (2019). Exercise Training in Older Adults, What Effects on Muscle Force Control? A Systematic Review of Randomized Clinical Trials. Arch. Gerontol. Geriatr..

[28] Aczel B., Bago B., Szollosi A., Foldes A., Lukacs B. (2015). Is It Time for Studying Real-Life Debiasing? Evaluation of the Effectiveness of an Analogical Intervention Technique. Front. Psychol..

[29] Klein J.G. (2005). Five Pitfalls in Decisions about Diagnosis and Prescribing. Br. Med. J..

[30] Croskerry P. (2013). From Mindless to Mindful Practice—Cognitive Bias and Clinical Decision Making. N. Engl. J. Med..

[31] Reilly J.B., Ogdie A.R., Von Feldt J.M., Myers J.S. (2013). Teaching about How Doctors Think: A Longitudinal Curriculum in Cognitive Bias and Diagnostic Error for Residents. BMJ Qual. Saf..

[32] Brush J.E., Lee M., Sherbino J., Taylor-Fishwick J.C., Norman G. (2019). Effect of Teaching Bayesian Methods Using Learning by Concept vs. Learning by Example on Medical Students’ Ability to Estimate Probability of a Diagnosis: A Randomized Clinical Trial. JAMA Netw. Open.

[33] Liu L. (2018). Occupational Therapy in the Fourth Industrial Revolution. Can. J. Occup. Ther..

[34] U.S. Census Bureau (2017). 2017 National Population Projections Tables: Main Series.

